# Relationship between Injection Rate and Contrast Enhancement on Three-dimensional Digital Subtraction Angiography of the Cerebral Arteries

**DOI:** 10.5334/jbsr.1619

**Published:** 2018-12-03

**Authors:** Satoshi Takagi, Naoyuki Hanasaki

**Affiliations:** 1 Hokkaido University, JP; 2 National Defense Medical College Hospital, JP

**Keywords:** cerebral arteries, contrast media, digital subtraction angiography, intra-arterial injections, three-dimensional image

## Abstract

**Objective:**

Three-dimensional (3D) digital subtraction angiography (DSA) is becoming a common technique for the assessment of the cerebral arteries. Nevertheless, the injection parameters for each artery are not standardized among institutions. The objective of this study was to analyze the relationship between injection rate and contrast enhancement on 3D DSA of the common carotid artery.

**Materials and methods:**

Twenty-four patients who underwent 3D DSA of the common carotid artery from June 2013 to March 2015 were included in this retrospective study. Contrast enhancement of each patient was analyzed for four cerebral arteries segments (A1, A2, M1 and M2) by measuring the average pixel value on the source rotational two-dimensional DSA images. Linear regression analysis was used to investigate the correlation between injection rate and contrast enhancement.

**Results:**

All four regression lines showed that a higher injection rate led to higher contrast enhancement. There was a significant relationship for the A1, A2 and M1 segments (P = 0.008, 0.03 and < 0.001) but not for the M2 segment (P = 0.13). The goodness-of-fit of the regression lines was high for the M1 segment (R^2^ = 0.63). However, as the size of the vascular lumen became narrower, the value for the A1 (R^2^ = 0.28) and A2 (R^2^ = 0.19) segments became lower.

**Conclusion:**

In 3D DSA of the common carotid artery, contrast enhancement of a relatively wide lumen could be optimized by adjusting the injection rate. However, it is difficult to optimize the contrast enhancement of a relatively narrow lumen only by adjusting the injection rate.

## Introduction

Three-dimensional (3D) digital subtraction angiography (DSA), which combines two-dimensional (2D) DSA and gantry rotation, is becoming a common technique for the assessment and the treatment of the cerebral arteries [1,2,3,4,5,6,7,8,9,10].

In most studies of 3D DSA, the injection rate and the volume of contrast medium have been determined based on the arteries involved and the position of the catheter tip [1,2,4,5,6,7,8,9]. However, the injection parameters for each artery vary among institutions and are not standardized. Further, although there are reports of different injection parameters being used for different patients [3,10,11], the criterion used for determination of these injection parameters has not been clearly described. There has been a report on the relationship between the injection rate and image quality on 3D DSA when the contrast medium was injected from the internal carotid artery [12]. However, in that study, only relatively thick blood vessels (internal carotid artery, proximal A1 segment, and proximal M1 segment) were evaluated, and the distal relatively thin blood vessels were only assessed by eye. Furthermore, it is unknown whether there is a similar relationship when the contrast medium is injected from the common carotid artery.

At our institution, we have attempted to optimize the injection rate for each patient by assessing the contrast enhancement of 2D DSA images obtained just before 3D DSA images. Although it has been difficult to obtain constant contrast enhancement with this method, we thought that the relationship between injection rate and contrast enhancement on 3D DSA of the common carotid artery could be clarified by analyzing these datasets.

Therefore, the purpose of this retrospective study was to analyze the relationship between injection rate and contrast enhancement on 3D DSA when the contrast medium was injected from the common carotid artery and to compare it with the results of the previous study in which the contrast medium was injected from the internal carotid artery.

## Materials and Methods

### Patients

Twenty-four patients (eight men, 16 women) of mean age 61 (range 35–84) years who underwent 3D DSA of the common carotid artery from June 2013 to March 2015 and whose injection parameters were recorded were included in this retrospective study. The study population included 12 patients with a brain tumor, 11 with an aneurysm, and one whose findings were normal. In all cases, the presence of a vascular anomaly, such as a fistula or embolus, was excluded on computed tomography angiography performed before 3D DSA. Our institutional review board approved this retrospective study and waived the requirement for informed consent from patients.

### Image acquisition

All 3D DSA procedures were performed using a biplane angiographic suite (Artis zee BA Twin; Siemens AG, Erlangen, Germany). The parameters used were as follows: rotation time, 5 s; rotation angle, 200° with 1.5° increments resulting in 133 projections; acquisition matrix, 1240 × 960; and dose, 0.36 μGy/f. The contrast medium (Omnipaque 300, Daiichi Pharmaceutical, Tokyo, Japan) was injected using a 4-Fr diagnostic catheter into either the right or left common carotid artery via a power injector (Mark V; Medrad Inc., Pittsburgh, PA, USA). The injection rate ranged from 3.0 to 5.0 ml/s, and the delay time from injection to scanning was 2 or 3 s. The 3D DSA examination was started with the contrast medium filled to the catheter tip, and injection was continued until scanning was completed. The duration of injection was 7 or 8 s and the total injection volume ranged from 21 to 36 ml. All patients received an anxiolytic agent shortly before the examination to suppress movement. All patients were awake during the examination, and 3D DSA was performed while the patient was free breathing. No patient movement that could have affected the quality of the images occurred during the examination, and there was no need for motion correction with image processing.

### Image analysis

**Figure 1:**
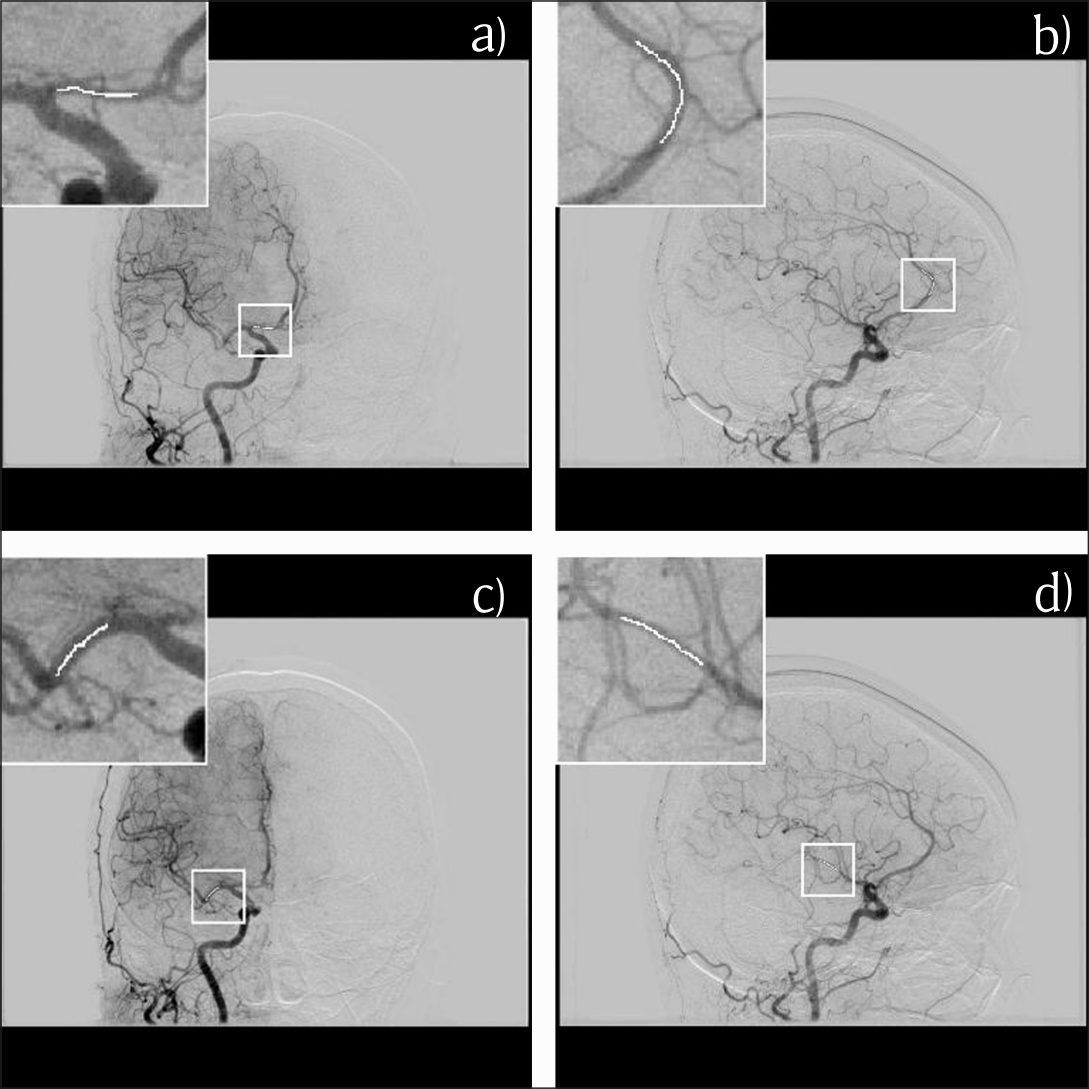
Measurement of contrast enhancement for the A1 **(a)**, A2 **(b)**, M1 **(c)** and M2 **(d)** segments on the source rotational two-dimensional digital subtraction angiography. The pixel value at the center of the projected vessel is extracted manually, and the average value on several images is calculated.

The quality of the 3D volume rendering (VR) image is affected by the reconstruction and rendering parameters [13]. Therefore, we analyzed the rotational 2D DSA images that were the source of the 3D DSA images. We defined four segments as the targets for analysis, i.e., A1 and A2 of the anterior cerebral artery and M1 and M2 (the branch leading to the angular artery) of the middle cerebral artery. Images on which the target artery was projected perpendicularly, those on which other arteries overlapped with the target artery, and those on which contrast enhancement of the background (brain parenchyma and vein) was not negligible were excluded. For each segment, the average values of a total of 1000 pixels at the center of the projected vessel on several images were manually calculated as contrast enhancement (Figure 1). A pixel value of 0 represented black and a value of 4095 represented white in the equipment. Therefore, the pixel value decreased with enhancement of the arteries.

### Statistical analysis

Linear regression analysis was used to investigate the correlation between the injection rate and contrast enhancement, and the coefficient of determination (R^2^) was calculated to assess the goodness-of-fit. All statistical analyses were performed using Statistical Package for the Social Sciences version 22 software (IBM Corporation, Armonk, NY, USA). A P-value < 0.05 was considered to be statistically significant.

## Results

**Figure 2:**
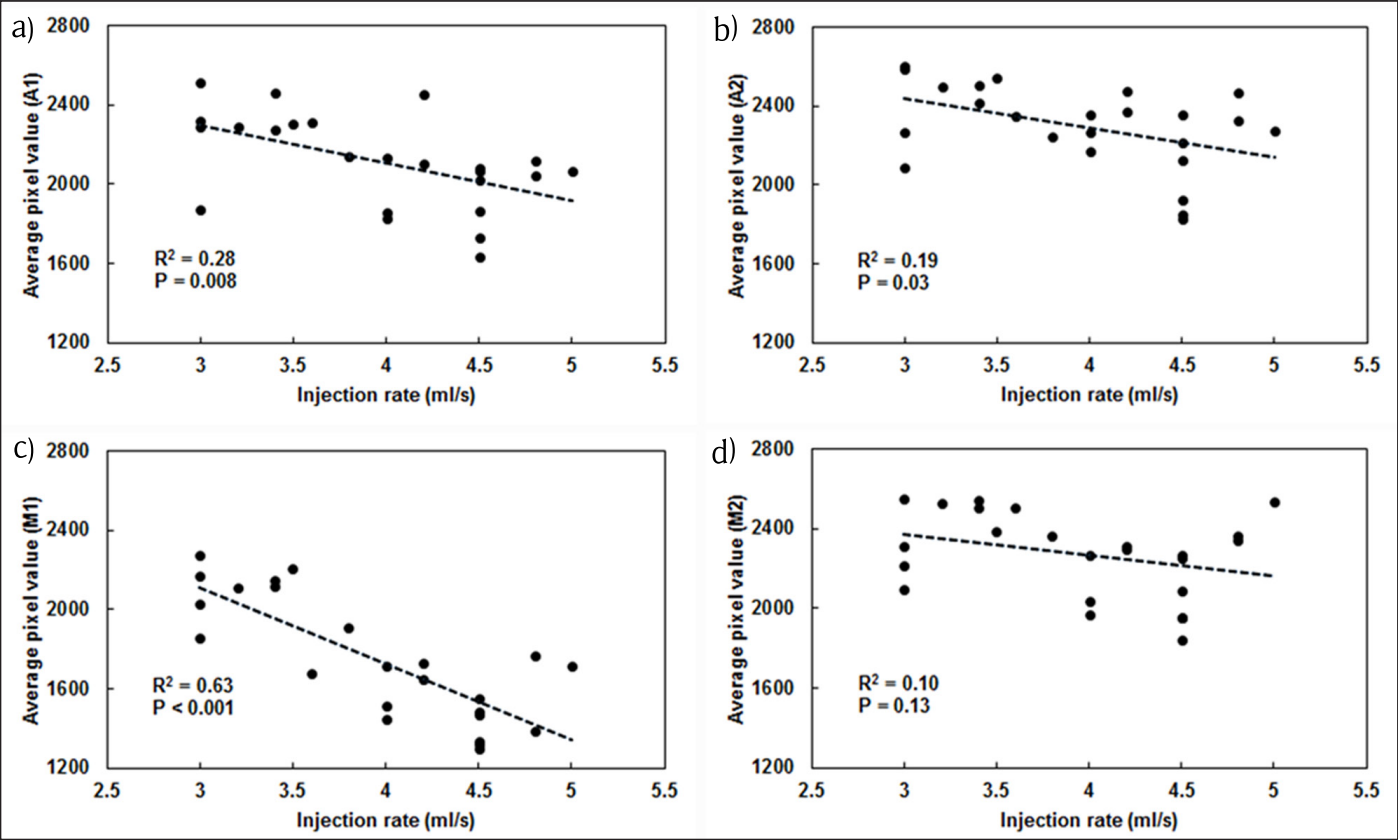
Relationship between injection rate and contrast enhancement for the A1 **(a)**, A2 **(b)**, M1 **(c)** and M2 **(d)** segments with straight lines fitted linear regression. Although the regression lines show that an increased injection rate lead to more contrast enhancement, there is no significant relationship for the M2 segment (d), which has the narrowest lumen size of the four segments.

The relationship between the injection rate and contrast enhancement for each segment is shown in Figure 2, with the straight line fitted by linear regression. All four regression lines showed that a higher injection rate led to higher contrast enhancement. There was a significant relationship for the A1, A2 and M1 segments (P = 0.008, 0.03 and <0.001) but not for the M2 segment, which had the narrowest lumen of all the defined segments (P = 0.13). The goodness-of-fit of the regression lines was the highest for the M1 segment, which had the widest lumen (R^2^ = 0.63). However, as the size of the vascular lumen became narrower, the value for the A1 (R^2^ = 0.28) and A2 (R^2^ = 0.19) segments became lower.

## Discussion

In this retrospective study, we determined the relationship between injection rate and contrast enhancement in 3D DSA when the contrast medium was injected from the common carotid artery. Although 3D DSA is generally used to evaluate vessel anatomy, relationship between injection rate and contrast enhancement has not been considered in sufficient detail. Three-dimensional DSA requires a larger volume of contrast medium than 2D DSA. Because over-injection increases the burden on the kidney, optimization of injection parameters is necessary for patient safety.

In the present study, in which the contrast medium was injected from the common carotid artery, the contrast enhancement in the A1 and M1 segments increased significantly as the injection rate increased. The contrast enhancement in the A2 and M2 segments also tended to increase in response to an increased injection rate, but the R^2^ values were very low. The results of this study are consistent with those previously reported when the contrast medium is injected from the internal carotid artery [12]. Therefore, the relationship between the injection rate and contrast enhancement could be considered to be the same whether the injection position was the internal carotid artery or the common carotid artery.

There was a significant relationship between injection rate and contrast enhancement with a high goodness-of-fit of the regression for the M1 segment, which had the widest lumen of all the evaluated segments. However, the R^2^ value became lower as the vascular lumen became narrower, and there was no significant relationship with regard to the M2 segment with the narrowest lumen. This relationship suggested a role of lumen size in contrast enhancement when using 3D DSA. Therefore, as the size of the lumen increases, although there are factors other than lumen size to consider, it could be helpful to adjust the injection rate for acquisition of the desired contrast enhancement. On the other hand, as the lumen size decreases, adjustment of the injection rate could become less useful for contrast enhancement. An increase in the injection rate for a relatively narrow lumen would not contribute to contrast enhancement, but only increase the burden on the kidney. We assume that contrast enhancement where the lumen size is relatively narrow is mainly affected not by the injection rate but by other factors, such as the patient’s weight, age, and hemodynamics.

The contrast enhancement on carotid angiography is influenced by the contralateral internal carotid artery via the anterior communicating artery and by the basilar artery via the posterior communicating artery. Moreover, the hemodynamics of the brain differ from disease to disease. Therefore, contrast enhancement is influenced by a complex set of factors. However, in this study, even when subjects with various diseases were included, a statistically significant relationship was found between the injection rate and contrast enhancement. Although it is conceivable that several factors may affect contrast enhancement, it seems likely that contrast enhancement would have been affected by the injection rate. Although constant contrast enhancement is not expected, to simplify the 3D DSA examination, it may be sufficient to fix the injection parameters when assessing a relatively narrow lumen that is less likely to be affected by the injection rate.

Unlike in 2D DSA, contrast enhancement on 3D DSA must be maintained during image acquisition. Therefore, it is important for optimization of injection parameters, including both the rate and duration of injection. Although the relationship between injection parameters and time to peak enhancement and peak enhancement on 2D DSA has been reported [14], the relationship between injection parameters and the duration of peak enhancement in 2D DSA has not been investigated. This relationship for each artery in 2D DSA could aid in determining the optimal injection parameters for constant contrast enhancement on 3D DSA.

Three-dimensional DSA images are finally reconstructed to derive 3D VR images. The rendering parameters greatly affect the characteristics of the 3D VR image, and the relationship between the contrast effects of 3D DSA and optimal VR parameters has been reported [13]. Inadequate rendering parameters can lead to over-estimation or under-estimation of lumen diameter. Therefore, constant contrast enhancement on 3D DSA is also useful for determining rendering parameters.

This study has some limitations. First, because the injection parameters for 3D DSA were recorded in only a small number of patients, the number of subjects included in this study was limited. Although a statistically significant relationship was found between injection rate and contrast enhancement, further investigations in larger numbers of subjects are required to assess this relationship in more detail. Second, although an influence of factors other than injection rate was suggested, patient characteristics at the time of examination were not recorded in our study. Therefore, investigation of other factors that might affect contrast enhancement was not possible. Third, although we were confident that use of various injection parameters in the same patient is the best way to clarify the relationship between injection parameters and contrast enhancement, this strategy is not realistic because of the increased injection volume and radiation exposure. Therefore, experiments in animals similar to those performed for 2D DSA are needed to clarify the relationship between various injection parameters and contrast enhancement in the same patient [14].

## Conclusion

In 3D DSA of the common carotid artery, contrast enhancement of a relatively wide lumen could be optimized by adjusting the injection rate. However, because of the effect of factors other than injection rate, as the lumen size becomes narrower, the effect of injection rate on contrast enhancement becomes less. It is difficult to optimize the contrast enhancement of a relatively narrow lumen only by adjusting the injection rate. Further studies on the relationship between contrast enhancement on 3D DSA and factors other than injection rate are required to optimize the injection parameters for constant image quality and patient safety.
